# Advanced Beamformers for Cochlear Implant Users: Acute Measurement of Speech Perception in Challenging Listening Conditions

**DOI:** 10.1371/journal.pone.0095542

**Published:** 2014-04-22

**Authors:** Andreas Buechner, Karl-Heinz Dyballa, Phillipp Hehrmann, Stefan Fredelake, Thomas Lenarz

**Affiliations:** 1 Department of Otolaryngology, Medical University Hannover, Hannover, Germany; 2 Cluster of Excellence hearing4all, Medical University Hannover, Hannover, Germany; 3 Advanced Bionics GmbH, European Research Center, Hannover, Germany; Northeastern University, United States of America

## Abstract

**Objective:**

To investigate the performance of monaural and binaural beamforming technology with an additional noise reduction algorithm, in cochlear implant recipients.

**Method:**

This experimental study was conducted as a single subject repeated measures design within a large German cochlear implant centre. Twelve experienced users of an Advanced Bionics HiRes90K or CII implant with a Harmony speech processor were enrolled. The cochlear implant processor of each subject was connected to one of two bilaterally placed state-of-the-art hearing aids (Phonak Ambra) providing three alternative directional processing options: an omnidirectional setting, an adaptive monaural beamformer, and a binaural beamformer. A further noise reduction algorithm (ClearVoice) was applied to the signal on the cochlear implant processor itself. The speech signal was presented from 0° and speech shaped noise presented from loudspeakers placed at ±70°, ±135° and 180°. The Oldenburg sentence test was used to determine the signal-to-noise ratio at which subjects scored 50% correct.

**Results:**

Both the adaptive and binaural beamformer were significantly better than the omnidirectional condition (5.3 dB±1.2 dB and 7.1 dB±1.6 dB (p<0.001) respectively). The best score was achieved with the binaural beamformer in combination with the ClearVoice noise reduction algorithm, with a significant improvement in SRT of 7.9 dB±2.4 dB (p<0.001) over the omnidirectional alone condition.

**Conclusions:**

The study showed that the binaural beamformer implemented in the Phonak Ambra hearing aid could be used in conjunction with a Harmony speech processor to produce substantial average improvements in SRT of 7.1 dB. The monaural, adaptive beamformer provided an averaged SRT improvement of 5.3 dB.

## Introduction

The performance of cochlear implant users continues to improve and many achieve good levels of speech perception, especially in quiet. Performance in noise however remains a challenge and manufacturers are constantly exploring different methods to improve it: Advances in the coding strategies used by the different devices, electrode design and the application of bilateral implants all contribute to improvements in performance [1]–[4]. However, technologies that have the ability to minimise the impact of noise in the incoming signal and improve the signal-to-noise ratio (SNR) have long been used in hearing aids and are now being explored in cochlear implants.

Cochlear implant manufacturers have used a number of different algorithms to reduce noise in the processing pathway such as Adaptive Dynamic Range Optimisation, used in recent versions of the Cochlear device [5] or ClearVoice, used in recent versions of the Advanced Bionics device [6]. Both these algorithms operate internally on a single-channel audio signal. More effective noise suppression techniques can be applied when signals from several microphones are available. Beamforming algorithms combine signals from multiple microphones to shape the directional response characteristics of the output. Rather than being equally sensitive to signals from all directions (omnidirectional), beamformers show highest sensitivity only for signals arriving from a limited range of angles. This directional selectivity can reduce the amount of noise drastically, if target and interfering signals are spatially separated in the environment. Despite being a standard feature in hearing aids for over a decade, the first beamforming algorithms for cochlear implant processors were commercially introduced in 2005 for users of the Cochlear Freedom sound processor [7]–[9].

Beamforming works by exploiting phase differences in the signal arriving at two or more spatially separated microphones from a particular direction of incidence. After applying an appropriate delay, the inputs can be subtracted from each other and different directionality patterns can thus be achieved. Beamformers can be used to produce a static pattern of directionality, where the point of maximum attenuation or null is fixed or an adaptive one, where the null follows dynamically the direction of noise incidence. That means adaptive systems have the advantage of being able to suppress moving noise sources.

In order for beamforming to work, a number of assumptions typically have to be met: the target should always be located within a narrow range on angles in front of the listener, and the target level should be identical at the two microphones. Well matched and calibrated microphones are essential and in reverberant rooms the performance has been shown to degrade, as both target and interfering noise become more spatially diffuse and consequentially more difficult to separate based on their directionality [10]–[13]. This limits the degree to which improvements measured in a highly controlled lab environment can be transferred to real life situations.

Directional microphones have been effectively employed in hearing aids to improve user performance in noise [14]–[17]. Ricketts and Henry (*2002*) compared fixed directional, adaptive, and omnidirectional microphone programs in 20 hearing impaired users of the Phonak Claro hearing aid (Phonak AG, Switzerland). They tested four noise conditions in a moderately reverberant room: (a) diffuse noise from 5 speakers at 75^0^ intervals, (b) noise from 2 speakers at 160^0^ and 200^0^ behind the listener, (c) noise from speakers at 70^0^ and 110^0^ to the side and (d) panning noise across all five speakers. Speech was always presented from 0° in front of the listener. The adaptive system was significantly better than the omnidirectional setting for all conditions, with a 2.9 dB improvement for sentences in diffuse noise. The fixed setting was only significantly better than the omnidirectional setting when the noise was presented from behind. The authors noted however that the fixed directional mode of the hearing aid used in the study was not optimised for diffuse noise, but noise coming from behind the listener. A systematic review of nine studies by Bentler in 2005 found evidence to support the effectiveness of directional microphones in current hearing aids [18]. McCreery (2012) identified seven paediatric studies, again indicating that directional microphones improve speech perception in controlled settings [19].

When hearing aids with adaptive directional microphone systems are fitted bilaterally, each system operates independently of the other. This situation was investigated by Mackenzie and Lutman: The authors found that although the adaptive systems worked independently, there were no detrimental effects, as measured by sentence recognitions scores in noise or quality ratings [20]. In fact, bilateral use of both the adaptive and the fixed system was consistently superior to the omnidirectional settings. However, in order to make the best use of a beamforming system using bilateral microphones the devices must communicate with each other [21]. The Phonak Ambra hearing aid (Phonak AG, Switzerland) implements such a system where the devices exchange audio data wireless between each other, effectively allowing beamforming to be performed using four microphones [22]. A different four-microphone spatial separation strategy was investigated by Kokkinakis et al. in five bilateral cochlear implant (CI) users [21]. The left and right audio signals were captured synchronously and processed together using a single processor, which was used to drive both CIs. However, the noise scenarios tested were limited, with speech shaped noise presented either from a single speaker at 90^0^ to the right or from three speakers placed at 30°, 60° and 90° to the right. Average improvements in percent correct scores over the unprocessed bilateral condition were 36% and 30% for the single and multiple noise sources respectively.

While several studies showed significant benefits of binaural beamformers for CI users [21], [23], [24], those studies were conducted using an experimental set-up that required a central body worn processing unit, which communicated continuously with both bilateral microphones as well as the CI system. The system investigated in our study was based on two hearing aids which can communicate with each other wirelessly and do not require a central processing unit. The major aim of this study was to compare the benefit of an adaptive monaural beamformer to the benefit of a wireless binaural beamformer for monaural CI users in a realistic set-up with noise presented from both, the frontal and rear, hemispheres.

### Study Objectives

This study investigated the performance of CI recipients using beamforming technology, as implemented in Phonak Ambra hearing aids, when combined with the Advanced Bionics Harmony speech processor (Advanced Bionics LLC, USA) in an experimental test setup. For this purpose the output signal of the Phonak Ambra hearing aid was galvanically coupled to the auxiliary input of the Harmony speech processor. The hearing aids used in this study implement a binaural beamformer using four microphones across the two hearing aids, producing a very narrow target beam and a monaural adaptive beamforming program called UltraZoom [22], [25]. The steering of the adaptive monaural beamformer is frequency specific and based on an approach proposed by Elko and Pong [26]. The two types of beamforming (adaptive monaural and binaural) were compared to an omnidirectional microphone setting.

The effect of adding ClearVoice (CV) as a post-processing stage to all three microphone settings was also assessed. CV is a single-microphone noise reduction algorithm implemented in the Advanced Bionics speech processor [6]. It acts on the signal after it has been band-pass filtered into the frequency ranges associated with the different CI electrode contacts, and is based on the assumption that the speech envelope is modulated while the noise envelope is stationary. The SNR is estimated separately in each frequency band, based on the modulation content, and bands with low estimated SNRs are attenuated. Attenuation of bands which are dominated by noise improves the overall SNR across all channels in the pulse stream transmitted to the CI user.

The study showed that both the binaural beamformer and the monaural adaptive beamformer implemented in the Phonak Ambra hearing aid could be used in conjunction with a Harmony speech processor to produce substantial average improvements in SRT. ClearVoice added a further benefit.

## Method

### Ethics Statement

The study protocol was reviewed and approved by a registered ethics board (Freiburger Ethik-Kommission International). After explanation of the study protocol and the risks and benefits of participating, all subjects signed a consent form before taking part.

### Subject Demographics

12 postlingually deafened subjects with an Advanced Bionics HiRes90K or CII cochlear implant were recruited randomly at the Cochlear Implant Center to participate in the study. All subjects were regular users of the HiRes120 processing strategy and had a minimum experience with their current strategy and processor of three months. All subjects had word scores of more than 15% for sentences in noise in the last clinical test. There were no criteria for the contralateral ear and five subjects were bilaterally implanted. If a subject was bilaterally implanted, the side with the better speech intelligibility was used, determined by the routine sentence test data collected in the clinic. The other side was turned off. The contralateral ear was plugged in subjects with residual contralateral hearing.

The mean age of implantation was 57 years, ranging from 30 to 72 years. The mean duration of deafness was nine years, ranging from zero to 30 years, based on the age when a telephone call was no longer possible till the age of implantation. The mean duration of cochlear implant use was three years, ranging from one to five years. Subject demographics are shown in table 1.

**Table 1:** Description of study participants.

Age at implantation in years	Duration of deafness in years	Duration of implant use in years	ClearVoice user?
71	16	2	rarely
62	17	4	yes
48	00	4	yes
30	30	1	yes
68	01	2	rarely
64	06	4	no
58	00	5	yes
58	16	4	no
58	06	2	no
57	00	2	yes
70	09	3	yes
72	01	2	yes

### Test Set Up

**Figure 1:**
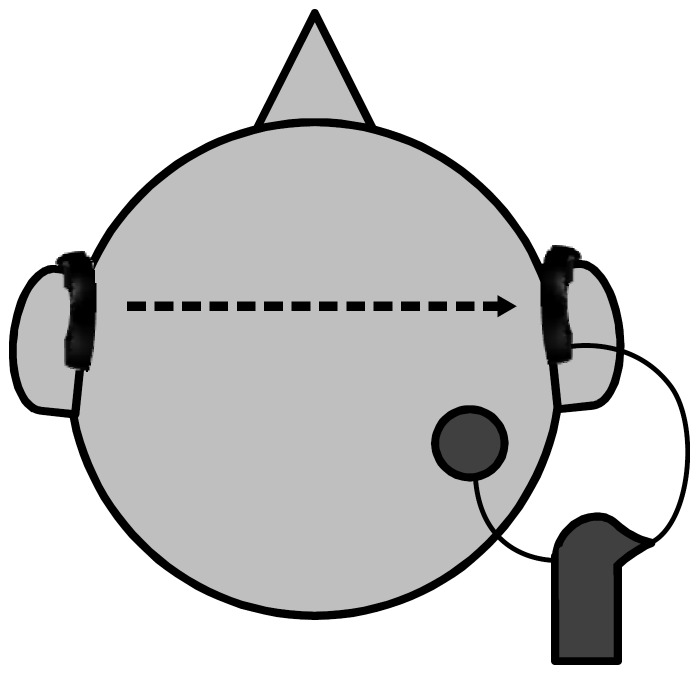
Set-up of hearing aids and cochlear implant processor. Subjects wearing Ambra behind-the-ear hearing aids on both sides communicating wirelessly; the hearing aid on the implanted side being connected to the cochlear implant speech processor.

Subjects were provided with an Ambra behind-the-ear (BTE) hearing aid which was placed ipsilaterally to the test side and connected to the speech processor, allowing the subjects to listen directly through the hearing aid. For the binaural beamformer condition a second hearing aid was placed on the contralateral ear, which was wirelessly linked to the other hearing aid connected to the speech processor (Figure 1). To connect the ipsilateral hearing aid with the speech processor, a technical modification had to be made: Instead of a hearing aid receiver a wire was attached to the output of the hearing aid to pick up the pulse-width modulated signal normally driving the miniature loudspeaker of the hearing aid. In order to generate an analogue audio signal from the hearing aid output as required by the speech processor, a low pass filter was applied using an LRC-circuit. This audio signal was delivered to the auxiliary input socket of the speech processor. With the hearing aid in omnidirectional mode, the frequency response of this experimental input configuration was confirmed to be equivalent to that of the regular omnidirectional BTE microphone of the speech processor.

Using the integrated pair of microphones of the modified hearing aid positioned at ear level, three different directional settings were programmed into the hearing aid:

1. Omnidirectional (omni): In this mode, the signal of the omnidirectional front microphone is transmitted to the speech processor without further processing.
2. The monaural adaptive beamformer (aBF): The aBF generates its directionality from one pair of microphones in the same device. It changes its directionality adaptively, based on the direction of noise.
3. The binaural beamformer (bBF): The bBF generates its directionality using two pairs of microphones on two separate ears. Thus, when using the bBF, a second hearing aid was needed on the contralateral ear. The two hearing aids were wirelessly linked and one was connected to the CI as described above.

### CI Signal Processing

Generally, CIs deliver time-staggered, amplitude-modulated electric pulse trains to an electrode array in the inner ear, where they excite the fibers of the auditory nerve. Current amplitudes are derived from the envelopes of a filter bank by the externally-worn speech processor and transmitted to the implanted circuit using a radio frequency link. For a typical user of the HiRes 120 coding strategy with 16 active implant electrodes, the number of filter-bank channels is 15, spanning a frequency range of approximately 250 to 8000 Hz [27].

ClearVoice is a noise reduction algorithm optionally available for users of HiRes 120. It applies attenuation to the output of each filter bank channel depending on the estimated *a posteriori* signal-to-noise ratios (SNRs), such that channels dominated by noise are dynamically attenuated. The background noise level is estimated using a voice-activity-dependent minimum tracking procedure. Channel-specific attenuations are determined based on a perceptually-motivated sigmoidal gain function. The highest possible attenuation per channel thus applied can be set to 6, 12 or 18 dB during fitting.

### Speech Processor Fitting

During testing, input to the processor was provided solely from the auxiliary input connected to the hearing aids. Before testing the speech processor was set to a sensitivity of 0. The volume dial allowed the subjects to change their M-levels globally by ±50%. Two programs were created: i) the clinical program without CV, ii) the same program with CV medium and with the most comfortable listening levels raised by 5% (as described in 6). The medium setting of CV was used, limiting the maximum amount of attenuation to 12 decibels (dB). Prior to each test condition, the subject was allowed to adjust the volume for optimal perception.

### Adaptive beamformer

**Figure 2:**
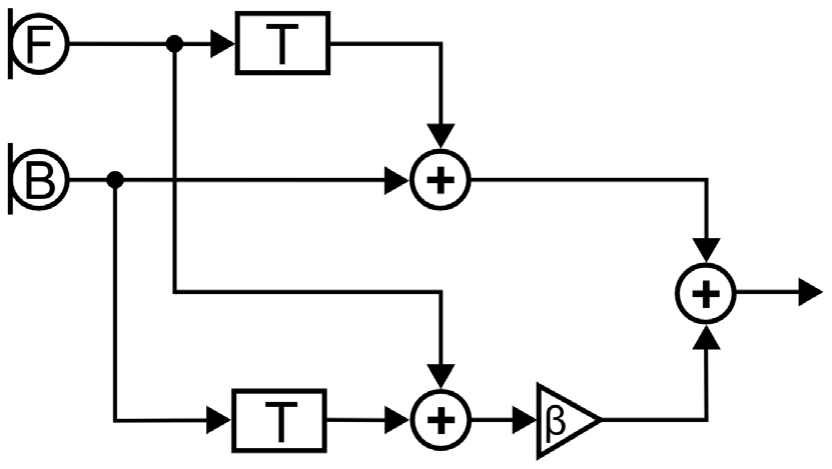
Flow-chart of the adaptive beamformer. The front (F) and back (B) microphone signals are added with a fixed delay to form the static cardioid beamformer. Afterwards, the beamformer outputs are added using a time-varying factor β for the adaptive beamformer. Not shown is the signal processing in different frequency bands.

The adaptive beamformer is based on the approach proposed by Elko and Pong [26] and consists of two signal processing steps in order to achieve the adaptive time-varying behavior as shown in Figure 2. In the first step, the signals from both omnidirectional microphones in the device are processed with two static cardioid beamformers with one cardioid facing 0° and the other one facing 180° direction. This is achieved by applying a constant time delay T to one microphone signal before subtracting it from the other. In the second step, the output of the back-facing beamformer is added to that of the front-facing one with a time-varying weighting factor β, which is adjusted to minimise the expected short-term power of the summation signal. As a result, the null of the adaptive beamformer is placed towards the direction with the highest noise power in the back hemispherewhile preserving signals from front. This processing is frequency-specific, such that multiple noise sources with non-overlapping frequency content can be cancelled simultaneously even when they are located at different spatial positions.

### Binaural beamformer

**Figure 3:**
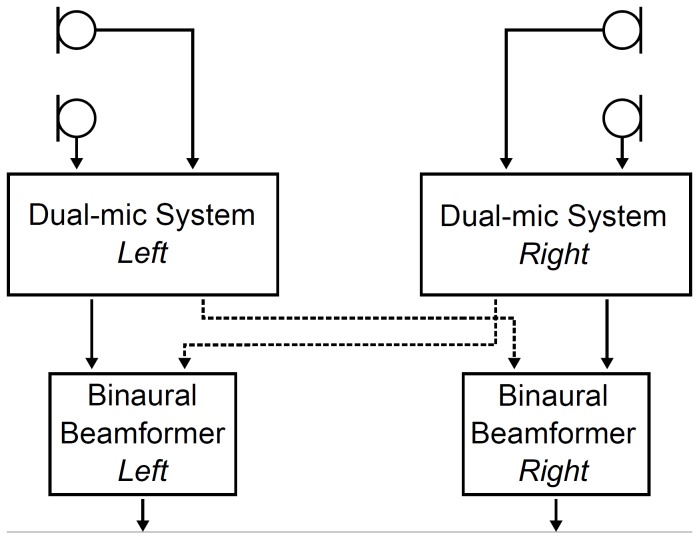
Flow-chart of the binaural beamformer. In the left and right device a dual-microphone beamformer is calculated and the audio signal is wirelessly transmitted to the opposite device yielding in the binaural beamformer.

The binaural beamformer requires two dual-microphone systems, one on each ear (Figure 3). First, a monaural beamforming algorithm is applied independently within each dual-microphone system. The two resulting audio signals are then transmitted wirelessly and concurrently to the respective contralateral device. There, they are combined with the contralateral dual-microphone output, effectively resulting in a 3^rd^ order (i.e. four-microphone) beamformer. The combination of signals across ears yields in a narrower and more focussed frontal and lateral beam pattern especially in the low-frequencies.

Both microphone systems attenuate noise from rear and lateral angles while maintaining the signal level from 0° front, thus yielding in an improved signal-to-noise ratio compared to an omnidirectional microphone system. Since the binaural beamformer has a more focussed beam to the front, i.e., more attenuation of surrounding noise, more improvement of the signal-to-noise ratio is achieved than with the monaural adaptive beamformer.

### Objective comparison of microphone directionality

**Figure 4:**
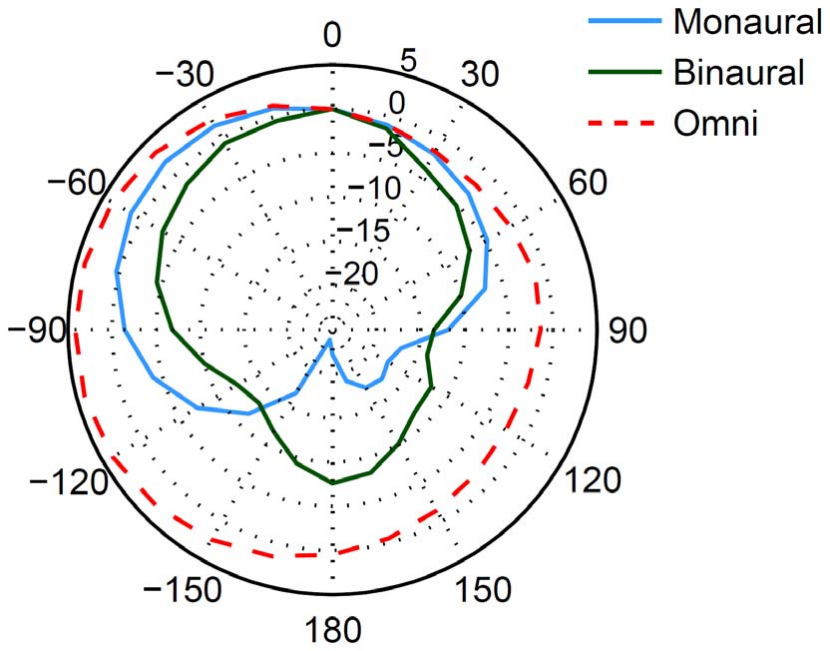
Spatial characteristic of the monaural and binaural beamformer. The polar plots are showing the microphone response for the omnidirectional microphone as well as monaural and binaural beamformers on a KEMAR dummy. Circles indicate the gain in decibels (dB) relative to the 0^0^ response.

A quantitative measure of microphone directionality was derived from polar plots obtained for a hearing aid on the left ear of a KEMAR artificial head in an anechoic room using a single noise source from varying angles (Figure 4). The radius in a given direction of the polar contour signifies the amount of attenuation of the processed signal from that direction relative to a signal from 0°. In order to obtain meaningful and comparable polar patterns, the adaptive beamformer was switched to a static mode for this particular measurement. For the lateral angles, the binaural beamformer attenuated sounds more than the monaural beamformer (by approximately 5 dB for a -60° angle, for example). Compared to the monaural beamformer, the maximum attenuation in the back hemisphere under anechoic conditions is less pronounced with the binaural beamformer. Nevertheless, the directivity index (i.e., the averaged attenuation from a microphone system for all angles relative to the attenuation for the front) reveals an overall attenuation of 3.4 and 4.6 dB for the monaural and binaural beamformer, respectively. In contrast, the omni directional microphone has a lower directivity index of −2.5 dB. In short, the binaural beamformer provides the highest directivity, i.e., a higher attenuation than the monaural beamformer, as characterised by a relative improvement in the directivity index of 1.2 dB.

As a further method to obtain an objective estimate of the prospective performance benefit for both beamforming algorithms,the relative attenuation of diffuse speech-shaped noise signals was determined in the particular spatial setup of this study: noise was simultaneously presented from the five noise speakers, and its speech-intelligibility weighted attenuation relative to a subsequent speech signal from the front was determined (Acoustical Society of America American National Standards for Calculation of Speech Intelligibility Index S3.5–1997). The SNR improvements thus estimated were 4.1 dB for the adaptive monaural and 7.6 dB for the binaural beamformer relative to the omnidirectional condition.

### Hearing Aid fitting

The hearing aid was programmed with a flat frequency response, providing 0 dB gain for all levels, and only the respective directional processing algorithm active. All other features were turned off. Furthermore, the hearing aid output level was matched to the input sensitivity of the CI system.

### Speech testing

Acute speech tests were carried out over the course of two sessions, one to six weeks apart, in a low-reverberant, sound-damped room. Each appointment typically lasted approximately two hours including two breaks. Six different conditions were tested and two were repeated at the following appointment, as shown in table 2. The order of conditions within each appointment, as well the order of the two appointments, was randomised. However, all conditions required for determination of the benefit of the aBF and its combination with CV were tested in a single appointment, while all conditions required to compare the aBF to the binBF as well as the maximal expected benefit of binBF and CV were covered in the other appointment. This distribution avoids additional spread of data due to differences in the physical or mental stage of subjects at the day of testing. Hence, appointment A investigated the difference between aBF and omni conditions and the additional effect of CV. Appointment B investigated the difference between aBF and bBF as well as measuring the benefit from bBF with CV.

**Table 2:** Conditions tested in each session.

Session A	Session B
Omnidirectional	Omnidirectional
Omnidirectional + ClearVoice	Adaptive Beamformer
Adaptive Beamformer	Binaural Beamformer
Adaptive Beamformer + ClearVoice	Binaural Beamformer + ClearVoice

**Figure 5:**
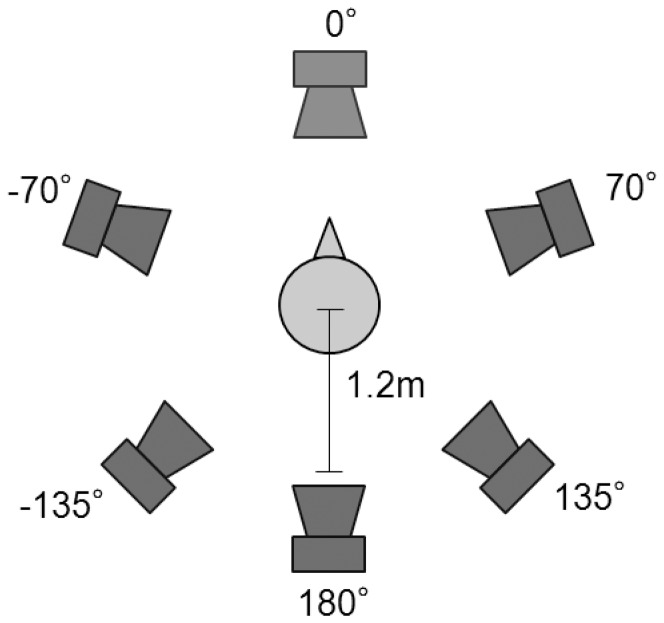
Speaker set up for speech perception testing. Six loudspeakers were positioned in a circle of 1.2^0^ and noise was presented simultaneously from the other five speaker locations.

The test set up was as shown in Figure 5. Six loudspeakers were located on a circle with a radius of 1.2 meters around the subject at angles of 0, ±70°, ±135° and 180°. The speech signal was presented from 0° while the noise was presented from ±70°, ±135° and 180°. Sounds were generated on a laptop connected to a RME Fireface 800 D/A converter, amplified with two Apart Champ Four amplifiers and finally presented via seven JBL Control 1 loudspeakers. To determine the speech intelligibility, the Oldenburg sentence test (OLSA) was used [28]. Sentences were presented in uncorrelated noise at an overall level of 65 dB SPL, with each loudspeaker calibrated for the same free-field presentation level. The OLSA noise is composed of an unintelligible, randomly-aligned superposition of all words in the test corpus, and thereby reflects the long-term spectrum of the speech material [29]. The speech level changed adaptively, depending on the answers of the subject, and the SNR determined where a speech intelligibility rating of 50% was scored (i.e., the speech reception threshold, SRT). Each test condition was assessed using a randomly chosen list and the consecutive list. The final individual SRT was calculated by averaging the two results for each condition. As training prior the test in the first condition, a minimum of four lists were presented.

### Study Design

A single-subject repeated measures design was used where each subject acted as their own control. Parametric statistics were applied. Data from appointment A was compared with a repeated measures two-way ANOVA, with the factors microphone directionality (omni vs. aBF) and noise reduction (CV on and off). Data from appointment B was compared using a one-way ANOVA to test for the effect of processing scheme only (omni, aBF, bBF, bBF+CV). Where a statistically significant effect was identified by the ANOVA, pairwise comparisons were made using two-tailed t-tests with Bonferroni corrected p-values. Following each ANOVA six pairs of data were compared, therefore a correction factor of 6 was applied and all p-values given in the text or graphs are the t-test p-value outcomes multiplied by 6. Means are quoted with 95% confidence intervals.

## Results

**Figure 6:**
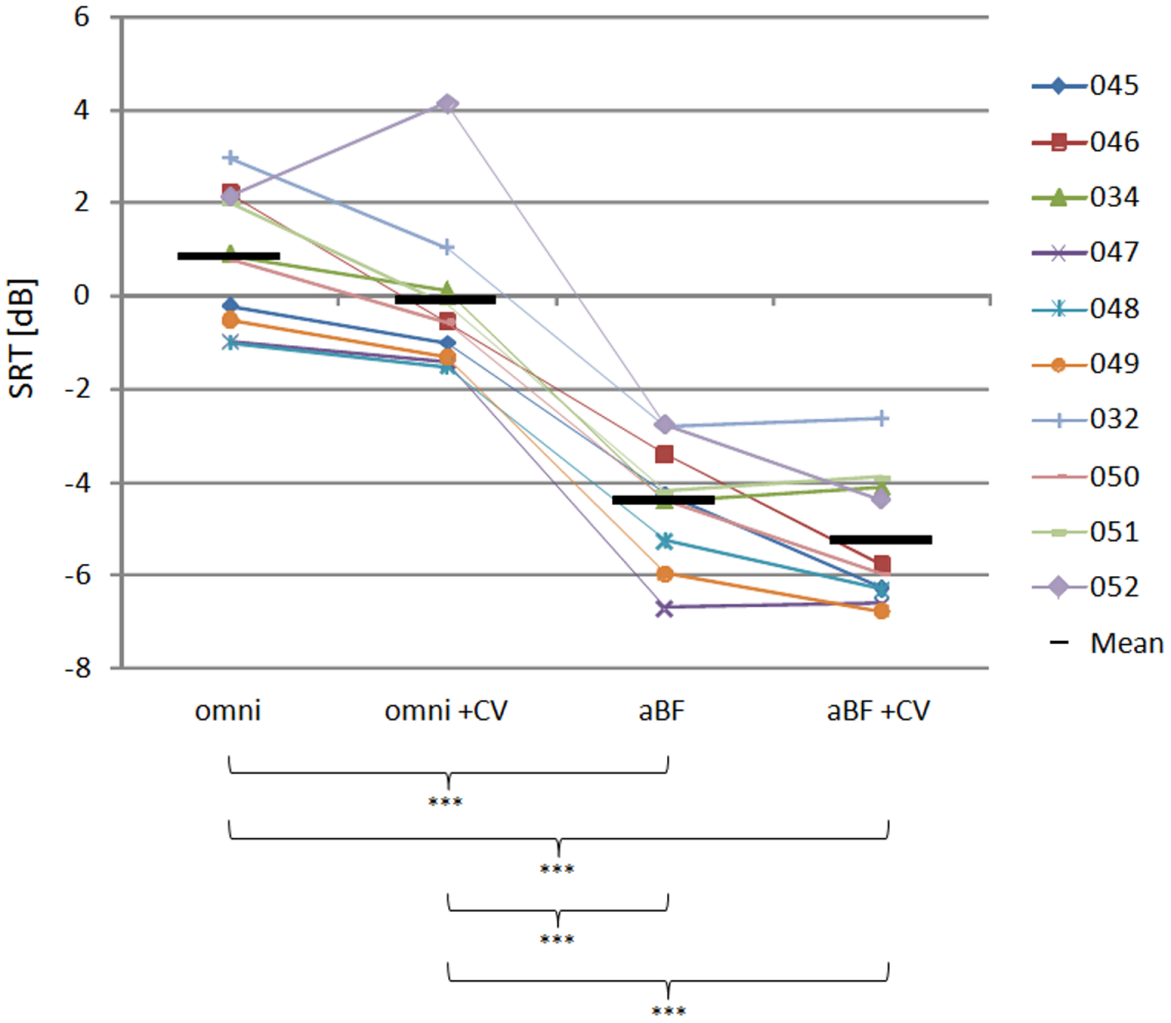
Mean and individual speech reception thresholds for the monaural adaptive beamformer test session. Speech reception thresholds (SRT) as measured for the OLSA sentences for each of the 10 subjects, in the four conditions tested during session A (omni – omnidirectional microphone; omni+CV – omnidirectional microphone with ClearVoice; aBF – monaural adaptive beamformer; aBF+CV – monaural adaptive beamformer mit ClearVoice). Short black lines indicate the mean of the study group; horizontal brackets indicate significant differences (*** at p<0.001).

Individual results from all 12 subjects are shown in Figure 6. Subject 44 did not attend for testing at appointment A and subject 43 was excluded because of significant residual hearing in the contralateral ear, which could not be successfully blocked with the ear plug. Statistical analysis was performed on the 10 remaining subjects, who had complete data sets for both sessions.

The results of the ANOVA are shown in table 3. The two-way ANOVA conducted for session A revealed a significant main effect of both microphone directionality (p<0.001) and noise reduction (p = 0.005) but there was no significant interaction between the two factors. The one-way ANOVA conducted for session B revealed a significant main effect of the processing scheme used (p<0.001). Post hoc testing conducted on both sets of data identified a number of significant differences of interest.

**Table 3:** Results of the ANOVA for Sessions A and B.

Factor	Processing Scheme	F Value	Degrees of Freedom (Hypothesis)	Degrees of Freedom (Error)	Significance
Session A					
Microphone directionality	aBF; omni	665.7	1	9	0.000
Noise reduction	CV; no CV	13.2	1	9	0.005
Interaction	omni; omni+CV; aBF; aBF+CV	0.03	1	9	0.859
Session B					
Processing scheme	Omni; aBF; binBF; binBF+CV	83.2	3	7	0.000

Figure 6 also shows the average SRT score for each condition tested in session A indicated by a short black line. The best performance was measured for the aBF+CV condition, with a large significant improvement over the omni condition of 6.1 dB±0.9 dB (p<0.001). The aBF condition without CV was also significantly better than the omni condition and the omni+CV condition (5.2 dB±0.7 dB (p<0.001), 4.3 dB±1.2 dB (p<0.001)).

**Figure 7:**
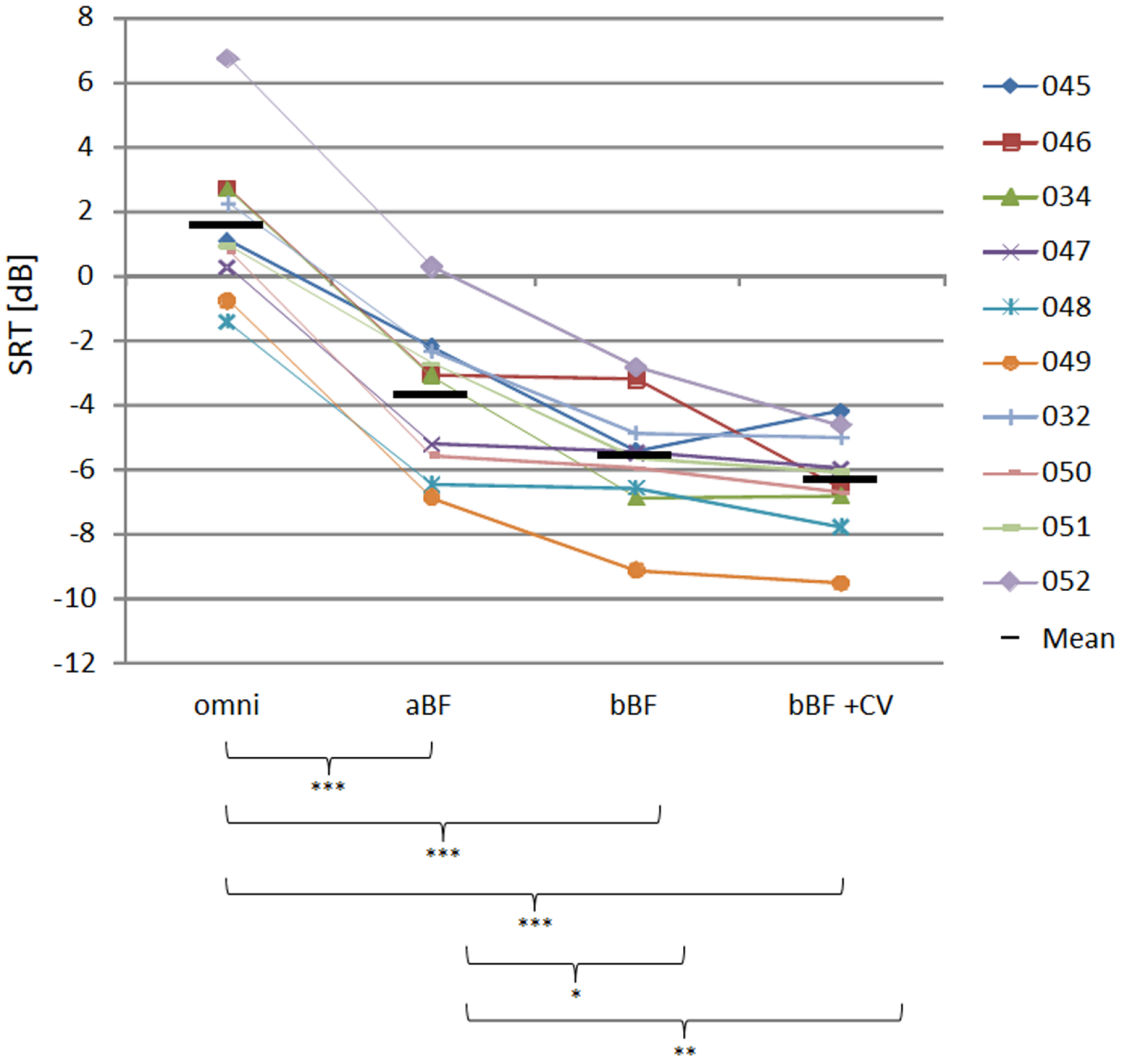
Mean and individual speech reception thresholds for the binaural beamformer test session. Speech reception thresholds (SRT) as measured for the OLSA sentences for each of the 10 subjects, in the four conditions tested during session B (omni – omnidirectional microphone; aBF – monaural adaptive beamformer; bBF – binaural beamformer; bBF+CV – binaural beamformer mit ClearVoice). Short black lines indicate the mean of the study group; horizontal brackets indicate significant differences (*** at p<0.001; ** at 0.001<p<0.01; * at 0.01<p<0.05).

Figure 7 shows the average SRT score for each condition tested in session B. The best performance in this session was achieved with the binaural beamforming plus CV condition, bBF+CV, with a significant improvement in SRT over the omni condition alone condition of 7.9 dB±2.4 dB (p<0.001). Both the aBF and bBF conditions alone were also significantly better than the omni condition (5.3 dB±1.2 and 7.1 dB±1.6, (p<0.001)) and were significantly different from each other by 1.9 dB±1.6 (p<0.05). However there was no significant advantage shown of adding CV to the bBF condition in the post hoc testing.

## Discussion

This study was conducted to investigate the performance benefit of monaural adaptive and binaural beamforming technology with an additional noise reduction algorithm in unilateral CI users. As still a significant number of CI users is unilaterally implanted and generally unilateral hearing conditions do not allow spatial release from diffuse background noise [30], the study focussed on unilateral CI use. Due to subject availability, a number of bilaterally implanted subjects were also recruited. For those subjects, the side they generally rely on less was always deactivated during the test sessions in order to mimic the situation of a unilaterally implanted user. Performance was only compared between unilaterally tested conditions and no comparison to the usually worn condition was done. Therefore any effect of bilateral interaction on the results can be excluded. During the test sessions, six loudspeakers were distributed on a circle around the subject; all but the front one presented noise to create a field with diffuse noise except for a narrow lobe from the front. This is a fairly realistic situation corresponding e. g. to a class room or lecture hall where the signal of interest comes from the front but background noise from both sides and rear.

The results of this study show that the use of beamforming technology significantly improved performance over the omni setting in diffuse noise in all conditions. The largest SRT improvement of 7.9 dB over the omni setting was observed for the bBF+CV condition. When looking at the effect of microphone directionality only, an SRT improvement of 7.1 dB was found for bBF compared to omni.

The SNR improvements estimated by the speech-intelligibility weighted noise attenuation were 4.1 dB for the adaptive monaural and 7.6 dB for the binaural beamformer, which was broadly consistent with the differences in directivity indices relative to the omni directional condition (5.9 and 7.1 dB) and the improvements found in the study subjects in appointments A and B (5.2 dB and 7.1 dB, respectively). Note that speech-intelligibility weighted attenuation was measured in the room and loudspeaker setup in which the subjects were tested, whereas the directivity index was measured with one single loudspeaker from different angles in an anechoic room. Different rooms and loudspeaker setups led to slightly different results between predicted SNR improvements and directivity indices. Nevertheless, both objective technical measures reflect more attenuation of noise with the binaural beamformer than with the adaptive beamformer, which is consistent with results obtained in subject testing.

In comparison to the adaptive monaural beamformer, the binaural beamformer proved to be significantly more efficient. The SRT difference between the two algorithms was 1.9 dB, consistent with in-house testing at Phonak where, in hearing-impaired hearing aid users, a difference between binaural and adaptive algorithms of 2.4 dB (p = 0.012) was shown [25]. The binaural beamformer derives the output signal from four microphones, which are arranged in pairs on either side of the head; the monaural beamformer is only based on the information of one microphone pair worn on a single side. Therefore the binaural beamformer has significantly more spatial information available and thus yields better suppression of spatially distributed noise.Although a comparison of these results with other studies is difficult, some implications can be made: A 6 dB improvement was reported by Wolfe et al. using a monaural fixed directional microphone. However, there are key differences between the studies [9]. The authors used a single multi-talker babble noise source directly from the side only rather than a diffuse noise pattern as was used here; the use of diffuse noise creates a more realistic and challenging situation, especially with the positioning of the noise speakers in the frontal hemisphere [24]. However, in the Wolfe study the results were compared to a standard microphone setting which is already moderately directional, thereby reducing the amount of benefit that can be expected from adding the beamformer [31], [32]. The SRT improvement of 7.1 dB reported here for the bBF condition compares favourably.

In the studies reported by Brockmayer et al [33], Hersbach et al [7] and Spriet et al [8], adaptive beamformers were tested in a laboratory situation. A gain of 6.5 dB was reported by Spriet et al. for three loudspeaker diffuse noise with loudspeakers positioned only in the back hemisphere at ±90° and 180° and 13.4 dB for a single loudspeaker positioned at +90°. However, adaptive beamforming is known to be most effective at reducing noise from the sides and behind the listener, with the source signal to the front. Studies using a single noise source placed either behind or at the side of the recipient, are likely to show the greatest improvement, while the setup itself is not realistic at all. Hersbach et al. reported gains of 6 dB but with a moving noise source, roving between seven speakers positioned at 30° intervals between 90° and 270° behind the listener. None of the above mentioned studies used noise sources in the frontal hemisphere, thus better performance than in our setup could be expected. Some studies have attempted to test beamforming in more realistic conditions [33], [34]. With the R-SPACE set up, uncorrelated restaurant noise is presented simultaneously from eight speakers positioned at 45° intervals all around the subject, including from the direction of the speech signal. Using this configuration reduced the reported improvements to between 3.9 and 4.5 dB at 60 dB noise over the subject's standard setting. Taking into account the variations in the test setups as discussed above, the 5.2 dB SRT improvement reported for adaptive monaural beamforming in this study is a remarkable outcome.

The two-way ANOVA with the factors microphone directionality (omni vs. aBF) and noise reduction (CV on and off) revealed a significant main effect of 0.8 dB for CV which was independent from the microphone directionality. This means the CV benefit is additive to the beamformer benefit. The benefit is smaller than expected from previous studies[6]. This discrepancy may be explained by the test methodology. Due to the very good SRTs obtained in this particular study group, even in the omnidirectional baseline condition (0.8 dB SNR in session 1), CV operated in an unfavourable SNR regime throughout the experiments, i.e. very close to zero.

According to the experiences in the hearing aid field, the benefit of beamformers is probably smaller in real life than in laboratory studies. On one hand, limitations of the experimental designs used so far may explain this discrepancy. On the other hand, reported problems from directional microphone studies in the hearing aid field are not directly applicable to the world of CIs: CI users still usually have little or no residual hearing and as a consequence, receive only the processed signal; no unprocessed, acoustic signals through an ear-mould venting can disturb the hearing like in hearing aid subjects, which commonly reduces the benefit of directional microphones in conventional hearing aids [35].

In order for beamforming to be used effectively the CI user must have a good understanding of when to use the beamformer. The narrow directional pattern of the binaural beamformer allows to focus on a single target source located opposite the listener, eliminating sounds from the back, sides and even off-centre frontal angles. This may be the appropriate configuration for a face-to-face conversation in a noisy bar or restaurant. If, however, the target speaker was to move positions constantly as in the case of a typical round-table discussion, the use of the adaptive beamformer is likely to be more advantageous. In situations where speech and noise are not sufficiently spatially separated, a single-channel noise reduction algorithm such as CV can still improve the listening performance.

When speech intelligibility is very difficult due to background noise and the talker is located in front of the CI user, then switching to a beamforming program will be appropriate. In contrast, for speech in quiet, the everyday program with omnidirectional microphone characteristic should be selected. In reality however, very few users change programs from their everyday settings and are often not used to seeking out noisy situations, but avoiding them [33]. Thus, a classifier that can detect acoustic situations and switch between programs and pre-processing algorithms would be highly desirable [36]. In case of a unilaterally worn CI system the binaural beamformer would not be available. Therefore the classifier only has to differentiate between noisy and quiet situations and accordingly switch between no preprocessing and adaptive beamformer in combination with ClearVoice. In case of a bilateral system more sophisticated rules are required. Based on similarities or differences between the raw signals at the four microphones the classifier has to decide whether the narrow beam of the binaural beamformer or the wider beam of the monaural adaptive beamformer is more appropriate. In principle, the available computational power of modern hearing systems is sufficient for the required algorithms. For the realization of a bilateral system, several options are conceivable; a unilaterally implanted CI user may use a satellite system on the contralateral ear which could look like a small light weighted hearing aid. This satellite may wirelessly transmit the two microphone signals to the contralaterally worn CI behind the ear processor. The CI processor would then perform all processing which was distributed between the hearing aids and the processor in our set-up. In case of bilaterally implanted CI users each processor on either side may integrate the required functionality for transmission and processing of all for microphone systems. As bimodal use with a CI on one side and a hearing aid on the other get more and more common the binaural beam former may also be realised by communication between a hearing aid and a linked CI processor. But in case of bimodal and bilateral CI use, further investigation is required to compare the binaural performance with the binaural beamformer to binaural performance with monaural or no beamformer. Binaural users have generally access to spatial cues which may become reduced or distorted with a binaural beamformer; their own physiological capability of spatial release from masking in many everyday life situations may be superior to a technical solution (as outlined above) that potentially interferes with their awareness for the room situation. However, also for those subjects a binaural beamformer is still of interest when selected intentionally for certain situations but not based on a classifier.

## Conclusions

The study showed that already the adaptive beamformer implemented in the Ambra hearing aid in conjunction with a Harmony speech processor produced a substantial improvement of 5.2 dB in SRT in a challenging noise setting with speech weighted noise compared to the omni-directional microphone. The binaural beamformer even produced SRT improvements of 7.1 dB over the omni-directional microphone. An overall trend towards better performance with CV was observed. Improvements of CV were likely limited in this particular study group, at least in parts, by the adaptive test methodology and the very good SRTs obtained, even without beamformer. The challenging test set up with noise sources also in the frontal hemisphere, aimed to produce a more realistic scenario compared to other studies, where noise was presented from one single speaker or within the rear hemisphere only. Even though the gain of the single-sided adaptive beamformer was remarkable, the results demonstrate that the potential performance benefits of a four-microphone binaural beamformer in challenging hearing situations are still large.
